# An overview on recent advances in the synthesis of sulfonated organic materials, sulfonated silica materials, and sulfonated carbon materials and their catalytic applications in chemical processes

**DOI:** 10.3762/bjoc.14.253

**Published:** 2018-11-01

**Authors:** Hashem Sharghi, Pezhman Shiri, Mahdi Aberi

**Affiliations:** 1 Department of Chemistry, Shiraz University, Shiraz, Iran

**Keywords:** acid-catalyzed processes, biodiesel synthesis, sulfonated carbon materials, sulfonated industrial and laboratory products, sulfonated organic materials, sulfonated silica materials

## Abstract

This review article discusses the progress related to the synthesis and catalytic applications of sulfonated organic materials, sulfonated silica materials, and sulfonated carbon materials for industrial and laboratory products. These catalysts are widely used in acid-catalyzed processes. Most of these acid catalysts are eco-friendly, reusable, and stable. Moreover, the discovery of unique catalysts is vital for developing new, efficient, and reusable catalysts for industrial and laboratory applications. The aim of this review article is to review the recent studies (2014–2018) in the ﬁeld of the utility of sulfonated organic materials, sulfonated silica materials, and sulfonated carbon materials for developing acidic catalysts.

## Review

### 1. Introduction

Mineral acids (sulfuric acid, sulfonic acid, hydrochloric acid, phosphoric acid, and boric acid) as homogeneous catalysts were usually applied for the synthesis of chemical products of great industrial and laboratory importance [1].

Organic and industrial reactions are performed well by the homogeneous acid catalysts, but these procedures generate extensive amounts of toxic residues [1]. Tackling the new century increasing environmental concerns is an important tendency for the development of new methodologies in both developed and developing countries. In fact, the expansion of new approaches to meet the needs of modern societies without harming the environment has become a fundamental principle [2–4].

In this context, it is pertinent to note that the industrial transformations in the presence of mineral acids need expensive and corrosion-resistant equipment. These non-reusable homogeneous acid catalysts have to be neutralized after the reaction, as well [5–6].

Methanesulfonic acid (MSA) and *p*-toluenesulfonic acid are commercial strong acids with p*K*_a_-values of −1.9 and −2.8, respectively, which are regularly used as simplest and more usable catalysts in chemical reactions. MSA is almost completely ionized at a concentration of 0.1 M in an aqueous solution. The oxidative stability of organic compounds and metal ions in MSA aqueous solution is probably well recognized in the literature. Less corrosive and toxic effects and the lack of dangerous vapors make it safe to handle under normal conditions. MSA as a biodegradable chemical is decomposed within 28 days by living organisms and is part of the natural sulfur cycle. During its decomposition, only CO_2_ and sulfate are formed [7–10]. Also, like MSA, *p*-toluenesulfonic acid (*p*-TsOH) is non-oxidizing, low cost, and easy to handle. Its physical state is solid, making it easy to work with [11]. According to these benefits, sulfonic acids are used as novel catalysts in organic chemistry [12–16].

**Figure 1:**
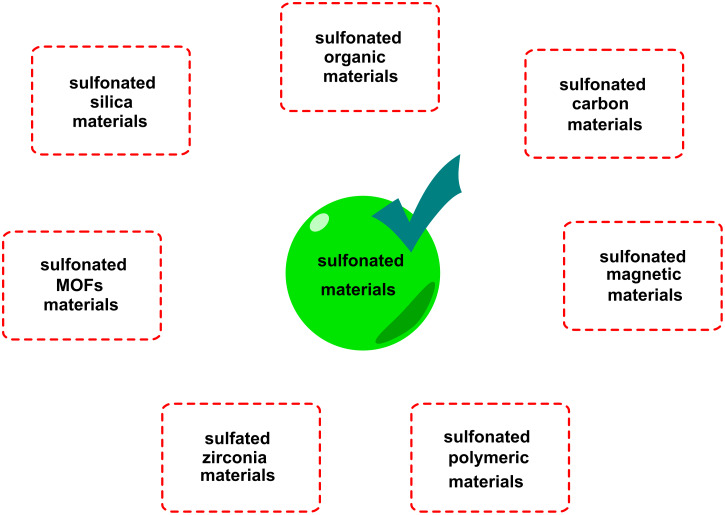
Different types of sulfonated materials as acid catalysts.

On the other hand, to reduce the toxicity and increase the efficiency, sulfonic acids are heterogenized on the various solid supports [17–19]. In fundamental, heterogeneous catalysis is interminably fascinating and perennially novel [20]. The few reports on sulfated solid supports are: sulfonated organic compounds [21], sulfonated silica materials [22–23], sulfonated carbon materials [5], sulfated zirconia [24], sulfated hybrid materials [25], sulfonated magnetic materials [26], sulfonated polymeric materials [27–28], sulfonated MOFs materials [29], and so on (Figure 1) [30–33].

Despite sporadic efforts, there is no detailed and updated report covering the diverse catalytic activities of sulfonated organic compounds, sulfonated silica materials, and sulfonated carbon materials in chemical processes. The current review discusses the uses of these catalysts for extensive reactions, including the synthesis of bis(indolyl)methane derivatives, β-amino carbonyl compounds, 14*H*-dibenzo[*a*,*j*]xanthene derivatives, 1,8-dioxodecahydroacridine derivatives, xanthene derivatives, pyrimido[4,5-*b*]quinoline derivatives, spiro-isatin derivatives, spiro-acenaphthenequinone derivatives, tetrahydrobenzo[*a*]xanthenone derivatives, tetrahydrobenzo[*a*]acridinone derivatives, 1-amidoalkyl-2-naphthol derivatives, 2*H*-indazolo[2,1-*b*]phthalazine-1,6,11(13*H*)trione derivatives, quinoline derivatives, bis-coumarin derivatives, 2*H*-indazolo[2,1-*b*]phthalazinetrione derivatives, triazolo[1,2-*a*]indazoletrione derivatives, tetrasubstituted imidazole derivatives, aromatic/aliphatic sulﬁde derivatives, and N-substituted pyrrole derivatives. These catalysts were used in cellulose hydrolysis, cellobiose hydrolysis, production of fatty acid ethyl ester, the transesterification of triglycerides with methanol, the etheriﬁcation of isopentene with methanol, the esteriﬁcation of palm fatty acid distillate with methanol, the dehydration of D-xylose into furfural, the production of ethyl acetate from ethanol and acetic acid, and the transesteriﬁcation of palm oil with methanol into biodiesel as well.

The efficacy of these sulfonated materials as novel catalysts is well-recognized with their benefits like ease of work-up, simple separation of catalysts from products, and economic usage in industrial procedures.

We believe that a comprehensive and systematic review of the established methodologies for preparing homogeneous and heterogeneous sulfonic acid based catalysts and their applications would be effective to a broad community of scholars working in chemistry laboratories and industries.

The present paper is intended to review briefly recent studies (2014 to 2018) concerning the synthesis of various organic and chemical products catalyzed by the titled catalysts.

### 2. Synthetic strategies of organic compounds containing sulfonic acid groups as a diverse class of catalysts and their uses in organic reactions

Organic compounds containing a sulfonic acid group have extensively considered to replace traditional mineral solid and liquid acids. These catalysts have been well developed and are one of the significant branches in organic chemistry, advanced materials, and nanotechnology. They have some unique properties including stability in air and aqueous environments, ease of handling, and even reusability [34].

Ionic liquids (ILs) have been extensively reported as green solvents in organic transformations, owing to their considerable properties such as the ability to dissolve a wide range of substances, very low vapor pressure, high thermal stability, recyclability, non-flammability, low volatility, and safety. These eco-friendly materials have been applied as a new category of catalysts in some organic reactions as well. Recently, new sulfonated ionic liquids and sulfonated solid salts have been prepared and used as efficient catalysts in various chemical reactions [35–37].

**Scheme 1:**
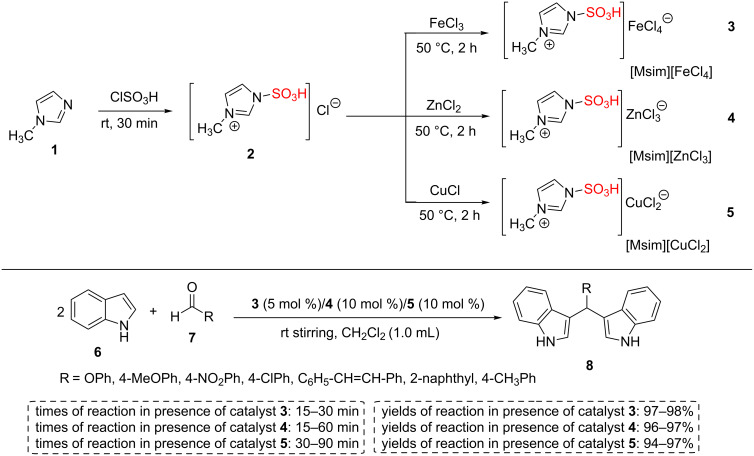
Synthetic route of 3-methyl-1-sulfo-1*H*-imidazolium metal chloride ILs and their catalytic applications in the synthesis of bis(indolyl)methane derivatives.

In an attempt, Gogoi et al. have reported new nanostructured sulfonated catalysts (3-methyl-1-sulfo-1*H*-imidazolium metal chlorides) containing both Lewis and Brønsted acidic sites **3**–**5** using the reaction of 3-methyl-1-sulfo-1*H*-imidazolium chloride (**2**) with FeCl_3_, ZnCl_2_ or CuCl in an inert atmosphere for 2 h. The 3-methyl-1-sulfo-1*H*-imidazolium metal chlorides [Msim][FeCl_4_] (**3**), [Msim][ZnCl_3_] (**4**), and [Msim][CuCl_2_] (**5**) were examined for the selective synthesis of bis(indolyl)methane derivatives **8** and the results showed that more acidic [Msim][FeCl_4_] catalyst **3** produces excellent yields of products **8** with only 5 mol % loading. However, 10 mol % of the less acidic [Msim][ZnCl_3_] (**4**) and [Msim][CuCl_2_] (**5**) catalysts were applied to obtain the desired products **8** with excellent yields (Scheme 1). A recyclability study indicated three consecutive runs with a similar efficiency [38].

**Scheme 2:**
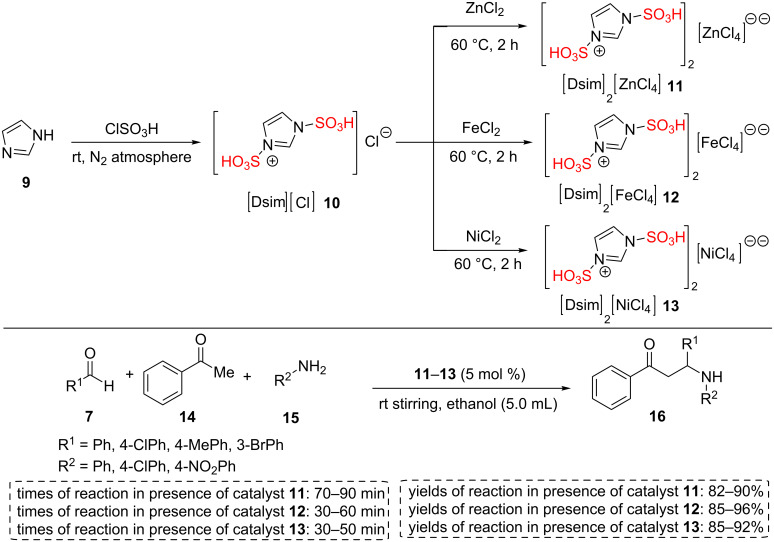
Synthetic route of 1,3-disulfo-1*H*-imidazolium transition metal chloride ILs and their catalytic applications in the synthesis of β-aminocarbonyl compounds.

Other researchers designed and synthesized a new number of 1,3-disulfoimidazolium transition metal chlorides including [Dsim]_2_[ZnCl_4_] (**11**), [Dsim][FeCl_4_] (**12**), and [Dsim]_2_[NiCl_4_] (**13**) as Brønsted-Lewis acidic solid materials. All these catalysts were reported as reusable and efficient catalysts for the multicomponent Mannich-type synthesis of β-aminocarbonyl products **16** in suitable times and yields (Scheme 2). To check the reusability of the catalysts, the reaction between benzaldehyde, aniline, and acetophenone in 5 mmol scale in ethanol was chosen. All catalysts were recycled three times using filtration of product solution in chloroform [39].

**Scheme 3:**
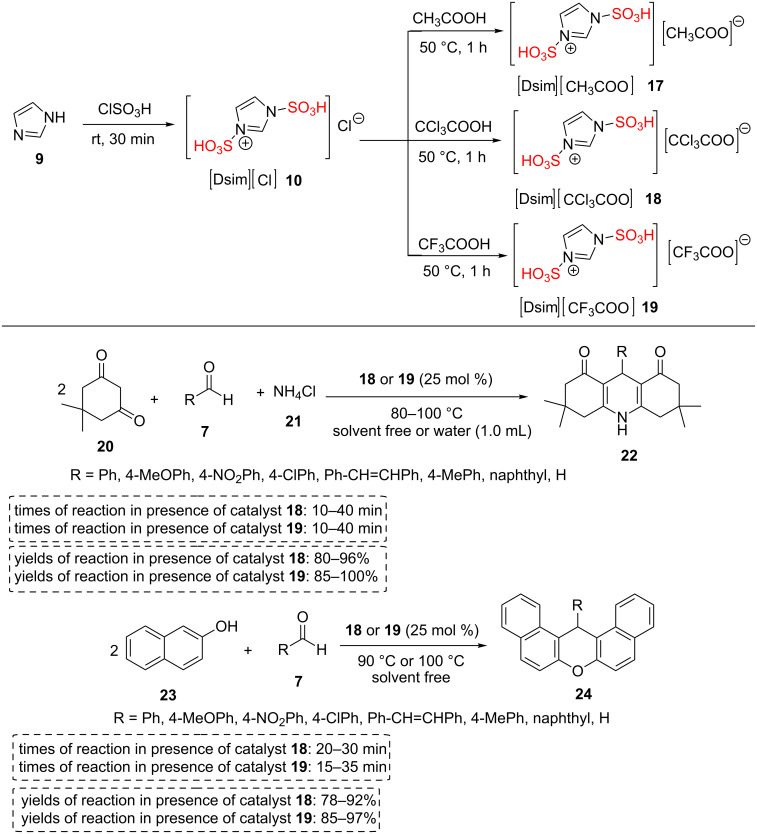
Synthetic route of 1,3-disulfoimidazolium carboxylate ILs and their catalytic applications in the synthesis of 14*H*-dibenzo[*a*,*j*]xanthene derivatives and 1,8-dioxodecahydroacridine derivatives.

1,3-Disulfo-1*H*-imidazolium carboxylate ILs [Dsim][carboxylate] **17**–**19** were synthesized using environmentally benign reactions between 1,3-disulfo-1*H*-imidazolium chloride [Dsim][Cl] (**10**) and three different carboxylic acids (CH_3_COOH, CCl_3_COOH, CF_3_COOH). The more acidic [DISM][CCl_3_COO] (**18**) and [Dsim][CF_3_COO] (**19**) ILs were utilized as recyclable, efficient, and eco-benign catalysts for the three-component one-pot condensations towards a variety of 1,8-dioxodecahydroacridine derivatives **22** and 14*H*-dibenzo[*a*,*j*]xanthene derivatives **24** in short reaction times under solvent-free or water medium with good to excellent yields (Scheme 3) [40].

Some results for these catalytic systems were summarized. The reaction between dimedone (**20**), aldehydes **7**, and ammonium chloride (**21**) produced excellent yields of 1,8-dioxodecahydroacridine derivatives **22** within 10–15 minutes at 80–100 °C using 25 mol % of [Dsim][CCl_3_COO] (**18**) or [Dsim][CF_3_COO] (**19**) ILs in absence of any solvent or in 1.0 mL of water. On the other hand, 1,8-dioxodecahydroacridine derivatives **24** were synthesized at 80–100 °C with good to excellent yields using 25 mol % of [Dsim][CCl_3_COO] (**18**) or [Dsim][CF_3_COO] (**19**) ILs in absence of any solvent. The [Dsim][CCl_3_COO] (**18**) and [Dsim][CF_3_COO] (**19**) ILs showed good recyclability and the catalysts were reused for three consecutive runs. In this case, the desired products were extracted by dry dichloromethane from the ionic liquid medium **18** or **19** and then the ionic liquids **18** or **19** were again applied for next runs.

**Scheme 4:**
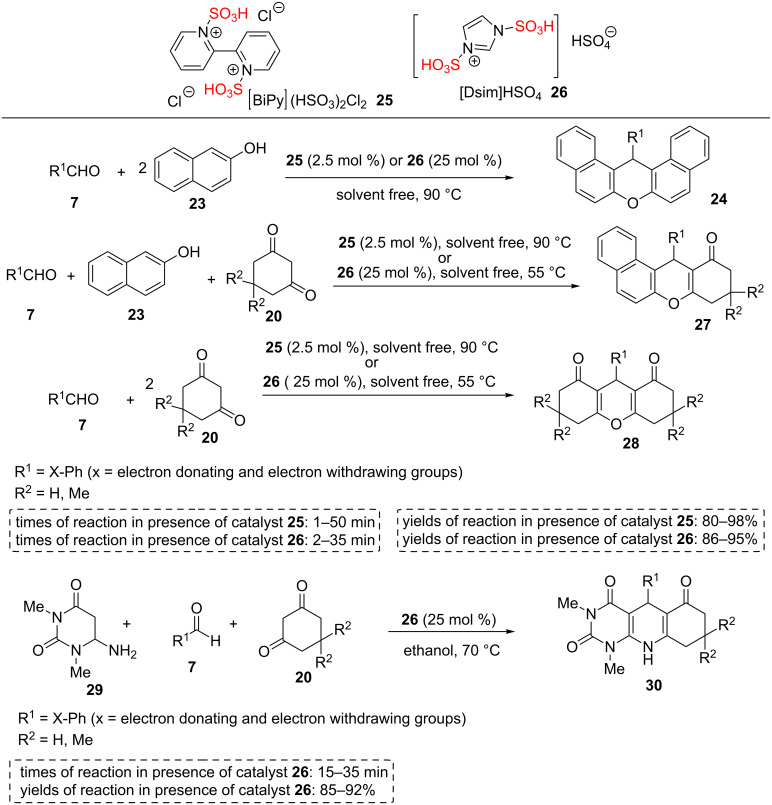
Synthetic route of [BiPy](HSO_3_)_2_Cl_2_ and [Dsim]HSO_4_ ILs and their catalytic applications for the synthesis of pyrimido[4,5-*b*]quinoline derivatives and xanthene derivatives.

Shirini et al. have reported a series of procedures for the synthesis of sulfonated materials and their applications for one-pot multicomponent reactions. This research group has reported a new route for the preparation of bi-SO_3_H ionic liquids based on 2,2'-bipyridine **25** using the reaction of chlorosulfonic acid and 2,2'-bipyridine as well as its application for the synthesis of the various xanthene derivatives **24**, **27**, and **28** [41]. In another study, the sulfonated imidazole **26** was prepared via the dropwise addition of chlorosulfonic acid to a stirred solution of imidazole in dry CH_2_Cl_2_ in an ice bath. In the next step, sulfuric acid 98% was added dropwise to the reaction mixture containing the sulfonated imidazole at room temperature to obtain 1,3-disulfo-1*H*-imidazolium hydrogen sulfate [Dsim]HSO_4_ (**26**) as a viscous pale yellow oil catalyst. [Dsim]HSO_4_ (**26**) has been also employed as a reusable and efficient catalyst for the one-pot multicomponent synthesis of various xanthene derivatives **24**, **27**, and **28** and pyrimido[4,5-*b*]quinoline derivatives **30** under mild and green conditions (Scheme 4) [42–44]. Easy preparation of the catalyst, easy reusability of the catalyst, easy handling, mild reaction conditions, low cost, excellent yields, short reaction times, and eco-friendly are some of the advantages of this work.

**Scheme 5:**
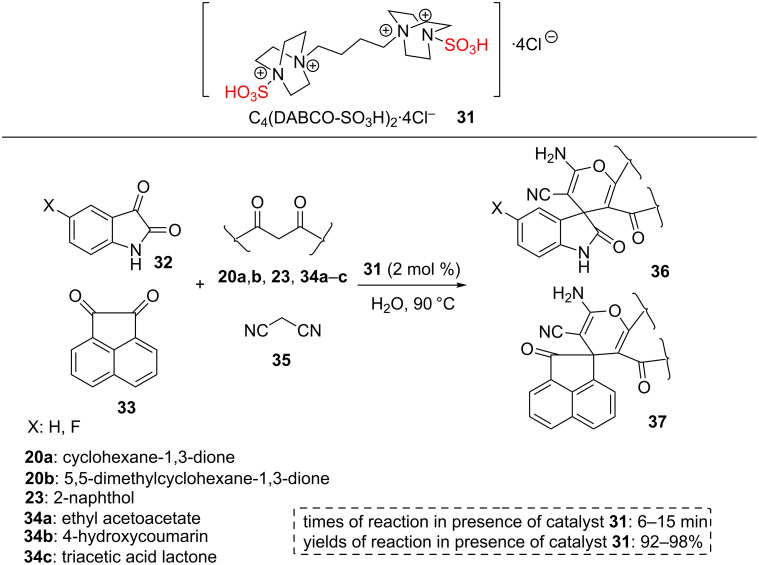
The catalytic applications of (C_4_(DABCO-SO_3_H)_2_·4Cl) IL for the synthesis of spiro-isatin derivatives and spiro-acenaphthenequinone derivatives.

**Scheme 6:**
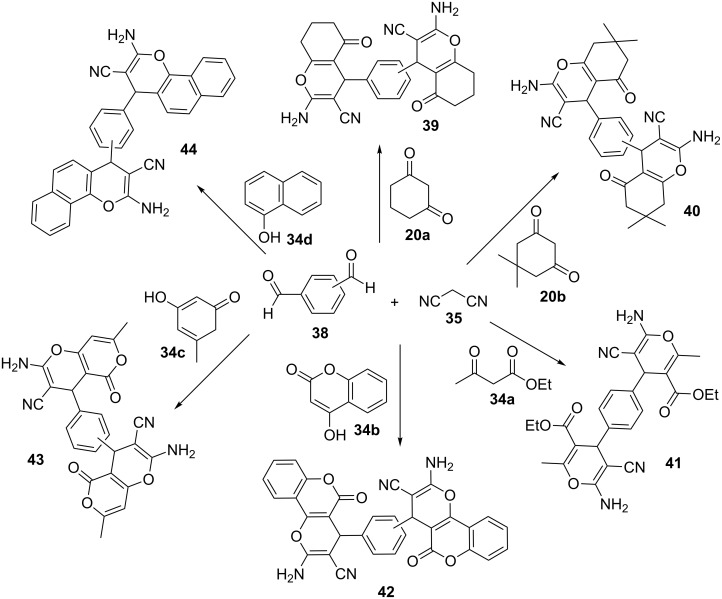
The catalytic applications of (C_4_(DABCO-SO_3_H)_2_·4Cl) IL for the synthesis of bis 2-amino-4*H*-pyran derivatives.

An efﬁcient, practical, and convenient strategy which is concerned with the construction of nanosized 4,4′-(butane-1,4-diyl)bis(1-sulfo-1,4-diazabicyclo[2.2.2]octane-1,4-diium) chloride (C_4_(DABCO-SO_3_H)_2_·4Cl, **31**) and its applications in the synthesis of spiro-oxindole derivatives **36** and **37** was described. C_4_(DABCO-SO_3_H)_2_·4Cl **31** acted as an efficient, cheap, and reusable nanocatalyst for synthesis of 2-amino-4*H*-pyran derivatives **36** and **37** from active carbonyl compounds (e.g., isatins **32**, acenaphthoquinone (**33**), and aldehydes **38)**, a variety of C–H activated acids (cyclohexane-1,3-dione (**20a**), 5,5-dimethylcyclohexane-1,3-dione (**20b**), 2-naphthol (**23**), ethyl acetoacetate (**34a**), 4-hydroxycoumarin (**34b**), triacetic acid lactone (**34c**), and 1-naphthol (**34d**)), and malononitrile (**35**) in water at 90 °C. Isatin (**32**) and acenaphthenequinone (**33**) were reacted with C–H activated acids **20a**,**b**, **23**, and **34a**–**c** and malononitrile (**35**) to form the corresponding spiro-isatin derivatives **36** and spiro-acenaphthenequinone derivatives **37** under mild and homogeneous conditions (Scheme 5). After this successful application, catalyst **31** was tested in the synthesis of bis(2-amino-4*H*-pyran) derivatives **39**–**44** via a one-pot multicomponent reaction of dialdehydes **38** (instead of isatin and acenaphthenequinone substrates), a variety of C–H activated acids **20a**,**b**, **23**, **34a**–**c** and malononitrile (**35**) under the same reaction conditions. The observations showed that bis(2-amino-4*H*-pyran) derivatives **39**–**44** are constructed in excellent yields during very short reaction times with a higher amount of the catalyst (4 mol %, Scheme 6). The recyclability of this homogeneous catalytic system was also studied by the reaction of isatin, malononitrile, and dimedone. After completion of the reaction, the reaction mixture was filtered and the same substrates were added directly to the filtrate solution containing the homogeneous catalytic system. There is no need to add solvent as well. The catalytic system worked for nine runs without considerable loss in its activity [45].

In 2017, the synthesis of *N*,*N*-disulfo-1,1,3,3-tetramethylguanidinium carboxylate ILs **47a**–**c** through reactions between *N*,*N*-disulfotetramethylguanidinium chloride (**46**) with three carboxylic acids (AcOH, CCl_3_COOH, and CF_3_COOH) in hexane at 60 °C for 45–60 min was achieved and reported. The chemical structures of new -SO_3_H functionalized ILs were confirmed by IR, ^1^H NMR, ^13^C NMR, and elemental analyses data. The NMR spectra provided evidence for resonating structures of *N*,*N*-disulfotetramethylguanidinium cations. The ^1^H NMR spectrum displayed all protons of two -NMe_2_ groups as a singlet in the region of 3–3.2 ppm. On the other hand, the carbon chemical shift of C=N appeared around 134.7 ppm and 119.9 ppm attributed to two types of the chemical environment of the C=N carbon [46].

**Scheme 7:**
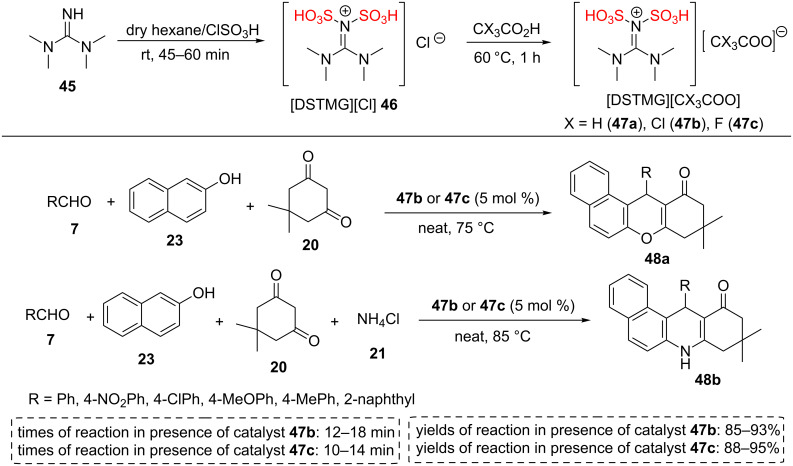
The synthetic route of *N*,*N*-disulfo-1,1,3,3-tetramethylguanidinium carboxylate ILs and their catalytic applications for the synthesis of tetrahydrobenzo[*a*]xanthenone derivatives and tetrahydrobenzo[*a*]acridinone derivatives.

The three-component synthesis of tetrahydrobenzo[*a*]xanthenone derivatives **48a** and the four component synthesis of tetrahydrobenzo[*a*]acridinone derivatives **48b** were performed with good to excellent yields under solvent-free conditions at 75–85 °C within short reaction times using the higher acidic/stable ILs containing trichloroacetate and trifluoroacetate anions **47b**,**c**. Following this method, various aromatic aldehydes bearing electron-withdrawing or donating groups (-NO_2_, -Cl, -OMe, -Me) **7** have been used to prepare the desired products in 85–95% yields. Aliphatic aldehydes produced complex mixtures of products using these homogeneous catalysts (Scheme 7). Signiﬁcantly, IL catalysts **47b**,**c** could be extracted from the reaction mixture for six consecutive cycles. In all runs, IL catalysts **47b**,**c** showed excellent catalytic activity. The FTIR spectra of two of these reused ILs after the 6th run and the fresh ILs have been used to prove the retention of their catalytic activity [46].

Khazaei et al. prepared 3-methyl-1-sulfo-1*H-*imidazolium tetrachloroferrate ([Msim]FeCl_4_, **3**) as a nanostructured catalyst via the reaction of 3-methyl-1-sulfon-1*H*-imidazolium chloride with dry FeCl_3_. After stirring the starting materials for 60 minutes at 70 °C, a dark green soiled salt was obtained in 98% yield. The catalyst was characterized by different analyses. The FE-SEM images exhibited that the particles of the catalyst are in nano size. According to XRD pattern, the crystallite size is at about 13.7 nm. The IR spectrum confirmed the presence of the O–H stretching of the -SO_3_H group at 2650–3550 cm^−1^ as well as the vibrational modes of N–SO_2_ and O–SO_2_ bonds at 1062 cm^−1^ and 1179 cm^−1^, respectively.

**Scheme 8:**
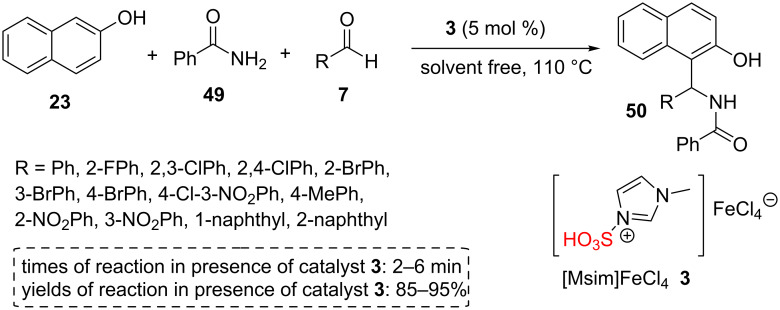
The catalytic application of 1-methyl-3-sulfo-1*H*-imidazolium tetrachloroferrate IL in the synthesis of 1-amidoalkyl-2-naphthol derivatives.

The catalyst **3** was found to be effective in the tandem reaction between β-naphthol (**23**), aromatic aldehydes **7**, and amide derivatives **49** at 110 °C under solvent-free conditions. The products were produced in very short reaction times and recrystallized in ethanol to give pure 1-amidoalkyl-2-naphthols **50** (Scheme 8). The reusability of the ionic liquid catalyst **3** was also studied. For this purpose, warm acetone was used to extract the products from the catalyst. The catalyst showed good catalytic activity for four successive runs [47].

**Scheme 9:**
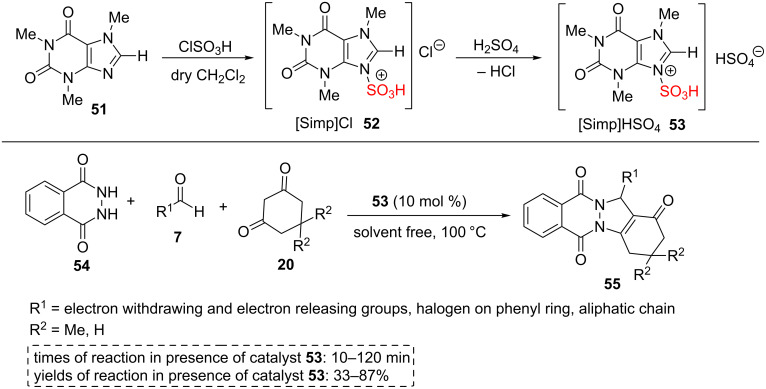
The synthetic route of 3-sulfo-1*H*-imidazolopyrimidinium hydrogen sulfate IL and its catalytic application for the synthesis of 2*H*-indazolo[2,1-*b*]phthalazine-1,6,11(13*H*)-trione derivatives.

Tayebee and co-workers prepared 3-sulfo-imidazolopyridinium hydrogen sulfate ([Simp]HSO_4_, **53**) as a new natural ionic liquid by the reaction between caffeine (**51**) as a natural, inexpensive, and available substance and chlorosulfonic acid for the first time. The authors proposed that the high Brønsted acidity of the catalyst arises mainly from hydrogen bonds between the two -SO_3_H groups. The catalyst **53** was studied by different analyses including FTIR, ^1^H NMR, ^13^C NMR, UV–vis, and ﬂuorescence spectra. Then, catalyst **53** was utilized for the synthesis of 2*H*-indazolo[2,1-*b*]phthalazine-1,6,11(13*H*)-trione derivatives **55** via a one-pot, three-component reaction of phthalhydrazide (**54**), aldehydes **7**, and dimedone (**20**) or cyclohexane-1,3-dione (for R^3^ = H, **20**) under solvent-free conditions (Scheme 9). To check the reusability of catalyst **53**, the reaction mixture was extracted with hot ethyl acetate. The residue was washed with hot ethyl acetate to afford the purely recycled catalyst. The catalyst **53** exhibited excellent reusability for 6 runs. Short reaction times, good to excellent product yields, a scaled-up synthesis and usage of the natural based ionic liquid as well as the high reusability of the catalyst are the advantages of this catalytic method [48].

Khaligh et al. [49] have synthesized two novel binuclear sulfonic-functionalized ionic liquids **56** and **57** with solvent-catalyst abilities for the synthesis of bis(indolyl)methanes **8**, **59**, and **60** under mild reaction conditions. The new ionic liquids **56** and **57** consist of a four-carbon spacer and an acidic anion. The structures of BBSI-Cl (**56**) and BBSI-HSO_4_ (**57**) were characterized using FTIR, MS, ^1^H and ^13^C NMR. The FTIR spectra of BBSI-Cl (**56**) and BBSI-HSO_4_ (**57**) displayed a broad peak at the range of 3500–3200 cm^−1^ related to stretching vibration of -OH groups in -SO_3_H and HSO_4_^−^ scaffolds and moisture. The peaks positioned at 3144, 3152, 2925, 2878 and 2854 cm^−1^ are related to C–H stretching vibrations of the aliphatic chain in BBSI-Cl (**56**) and BBSI-HSO_4_ (**57**). Two peaks due to C=C and C=N were observed at 1680 and 1540 cm^−1^. The bands at the range of 1200–1000 cm^−1^ are due to SO_2_ asymmetric and symmetric vibrations.

**Scheme 10:**
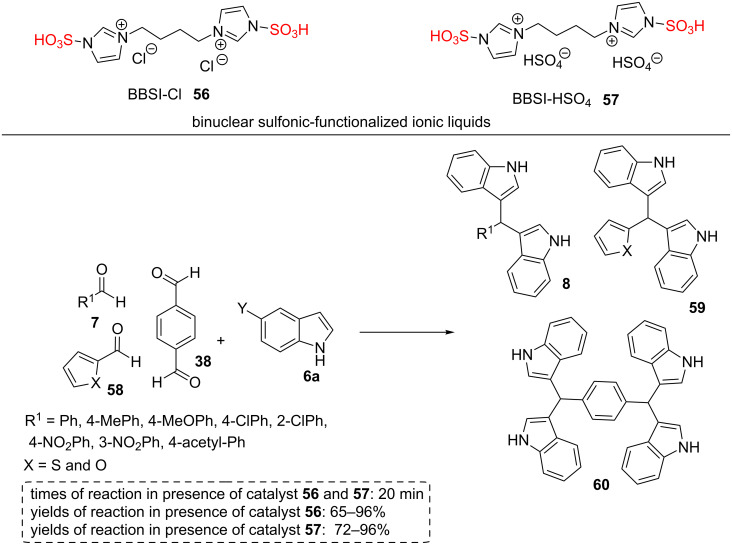
The results for the synthesis of bis(indolyl)methanes and di(bis(indolyl)methyl)benzenes in the presence of disulfonic-functionalized ILs.

A variety of aryl or heterocyclic aldehydes **7**, **38**, and **58** were reacted with indole or 5-bromo-1*H*-indole (**6a**) to synthesize the desired products **8**, **59**, and **60** in the presence of these solvent-catalyst ILs **56** and **57**. The catalytic efficiency of these two ILs (containing chloride or hydrogen sulfate counter anions) were screened in comparison with previously reported sulfonic acid-functionalized ILs derived from pyrazinium, piperazinium, benzimidazolium, and imidazolium as a cation part and chloride as an anion part (Scheme 10). Because of the presence of acidic anion, the catalytic activity of IL containing HSO_4_^−^ as an anion (BBSI-HSO_4_) was higher than IL containing Cl^−^ as an anion (BBSI-Cl). To increase the efficiency of the current procedure, the authors estimated the reusability of the solvent-catalyst ILs. The catalysts were removed with water from the reaction mixture. However, this way did not work in some cases, and the organic products were extracted from the ILs by nonpolar organic solvents. The organic products were extracted from the ILs by ethyl acetate or ether to give the recycled catalyst and the products. The remained ILs were concentrated and recharged with new starting materials for another run. The ILs showed the excellent catalytic activity for three consecutive runs. Finally, this research group explored the structure of reused BBSI-HSO_4_ (**56**) and BBSI-Cl (**57**) ILs after the third run by use of ^1^H NMR spectra. The ILs showed no noteworthy change in their structures. The advantages of these catalytic systems are using solvent-catalyst ILs, mild reaction conditions, diverse products, short reaction times, good reusability, good to excellent yields, and producing bis-products [49].

Amarasekara and co-workers described the effect of using sulfonic acid group functionalized ILs as catalysts in the hydrolysis of cellulose [50]. After this study, this research group reported that these sulfonic acid group functionalized ILs can also be applied in aqueous phase [51]. On the other hand, it has been proved that adding a catalytic amount of metal salts can slightly increase product yields [52]. So this research group prepared 1-(3-sulfopropyl)-3-methyl-1*H*-imidazolium chloride acidic IL **63** and the catalytic activity of this IL with or without a range of metal chloride salts was explored in the hydrolysis of cellulose in water. The best result was observed when Mn^2+^ was used in aqueous media at 170 °C; without using Mn^2+^, 28.7% of product was produced [52].

**Scheme 11:**
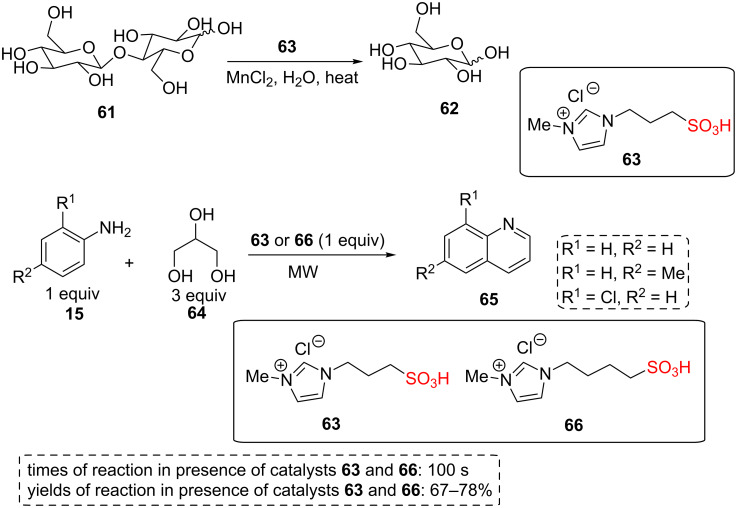
The catalytic applications of 1-(1-sulfoalkyl)-3-methylimidazolium chloride acidic ILs for the hydrolysis of cellobiose and the synthesis of quinolones.

In another study by this research group, the combination of this IL with manganese(II) chloride as a co-catalyst also exhibited excellent yield in cellobiose (**61**) hydrolysis in dilute aqueous sulfuric acid (Scheme 11). The authors found that the highest enhancement in the yield of the product happens at 60 °C. This may be owing to this fact that a weak interaction between manganese(II) chloride and cellobiose generates the co-catalytic effect. The interactions of MnCl_2_ with -OH groups and other oxygen atoms of cellobiose were confirmed by IR spectroscopy [53].

In another study, 1-(3-sulfopropyl)-3-methyl-1*H*-imidazolium chloride (**63**) and 1-(4-sulfobutyl)-3-methyl-1*H*-imidazolium chloride (**66**) ILs were used as excellent catalysts and the reaction medium for microwave synthesis of quinoline derivatives **65** from substituted anilines **15** and glycerol (**64**, Scheme 11). Some advantages of these catalytic systems include: elimination of oxidizing agents, ease of isolation of products, very short reaction times (only 10 s), and better yields. The use of glycerol as a starting material is another important advantage because it is the main byproduct in the biodiesel industry and the application of renewable feedstocks for the preparation of suitable chemicals and intermediates is of current interest [54].

### 3. Synthetic strategies of SiO_2_ and functionalized SiO_2_ containing sulfonic acid groups as a diverse class of catalysts and their uses in organic reactions

Nowadays, nanotechnology utilizing substances in the nanometer scale has attracted increasing attention in many fields including adsorbent, optical devices, water purification, drug delivery, and catalysis. Silica nanoparticles with different structures have extensively investigated due to their simple preparation and diverse industrial applications. In addition, SiO_2_ nanoparticles with high surface area commonly are the first option for heterogenizing the homogeneous catalysts. These solid supports have greatly functionalized with various functional groups [55]. In this regard, different functionalized SiO_2_ containing sulfonic acid groups as novel acid catalysts were employed in different synthetic and multicomponent reactions and some of them were mentioned below.

**Scheme 12:**
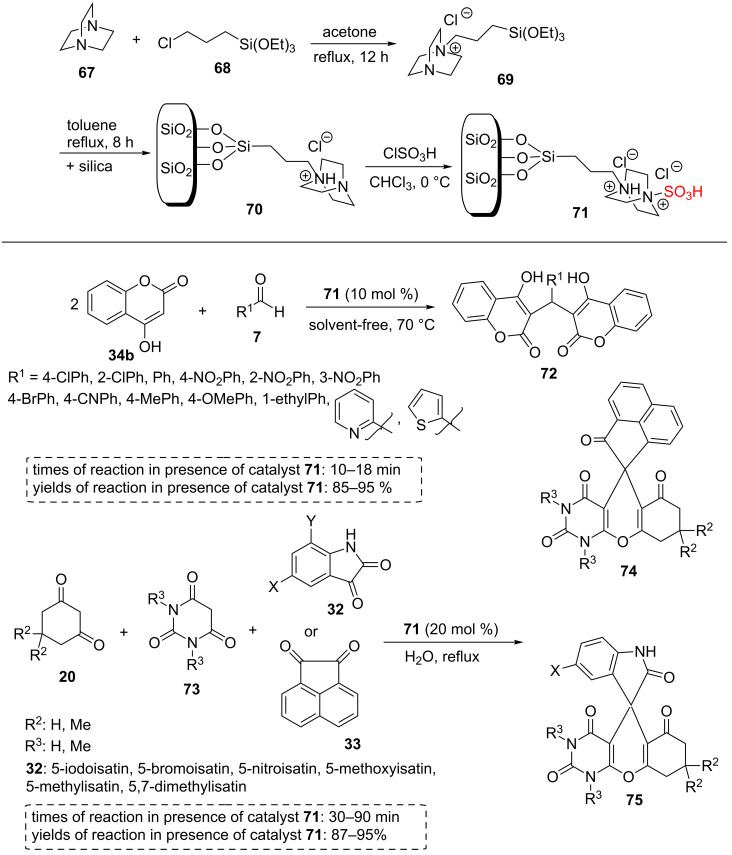
The synthetic route of immobilized 1,4-diazabicyclo[2.2.2]octanesulfonic acid chloride on SiO_2_ and its catalytic applications for the synthesis of bis-coumarins and spiropyrans.

Moosavi-Zare et al. immobilized 1,4-diazabicyclo[2.2.2] octanesulfonic acid chloride on SiO_2_ as a nanostructured heterogeneous catalyst. The silica-bonded 1,4-diazabicyclo[2.2.2]octanesulfonic acid chloride catalyst **71** was prepared in some steps as demonstrated in Scheme 12. Initially, **69** was formed using the reaction between 1,4-diazabicyclo[2.2.2]octane (**67**) and (3-chloropropyl)triethoxysilane (**68**) in refluxing acetone for 12 hours. In the next step, **70** was produced using the reaction between SiO_2_ and **69** in refluxing toluene for 8 h. Finally, silica supported 1,4-diazabicyclo[2.2.2]octane **70** was reacted with ClSO_3_H in cold chloroform to give new -SO_3_H functionalized SiO_2_.

The authors studied its catalytic behavior in the synthesis of bis-coumarin derivatives **72** using a solvent-free reaction of aryl aldehydes containing electron-donating and electron-withdrawing substitutions **7** with 4-hydroxycoumarin (**34b**) at 70 °C within short reaction times. All aromatic aldehydes **7** reacted with 4-hydroxycoumarin (**34b**) to form bis-coumarins bearing electron-donating groups, electron-withdrawing groups, and halogens **72** in very short reaction times and in high yields.

In another published article by this research group, silica-supported sulfonated 1,4-diazabicyclo[2.2.2]octane **71** was used for the one-pot tandem Knoevenagel–Michael cyclization reaction between isatin derivatives **32** or acenaphthenquinone (**33**), barbituric acid derivatives **73**, and 1,3-dicarbonyl compounds **20** to afford spiropyran derivatives **74** and **75** in aqueous media under reflux conditions (Scheme 12) [56]. The particular features of these protocols are short reaction times, high reaction yields, mild reaction conditions, and diverse desired products. The silica-bonded sulfo-1,4-diazabicyclo[2.2.2]octane chloride **71** was introduced as a highly efficient, reusable, general, and nanostructured catalyst for the synthesis of bis-coumarins **72** and spiropyrans **74** and **75** (Scheme 12) [57].

**Scheme 13:**
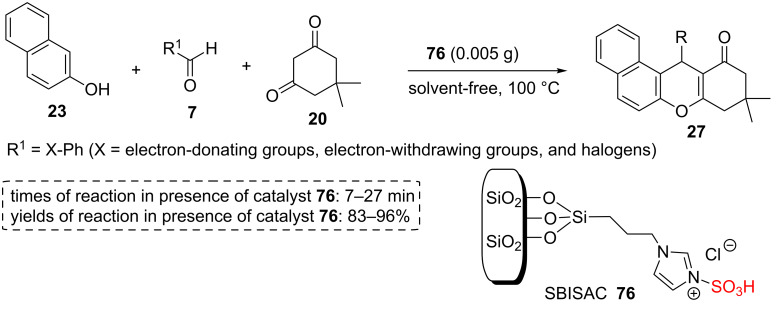
The catalytic application of a silica-bonded sulfoimidazolium chloride for the synthesis of 12-aryl-8,9,10,12-tetrahydrobenzo[*a*]xanthen-11-ones.

A silica-bonded sulfoimidazolium chloride **76** was prepared nearly similar to the previous example. After producing silica supported imidazole derivative, it was sulfonated by the reaction of supported imidazole with ClSO_3_H in cold chloroform to give new -SO_3_H functionalized SiO_2_. To screen the scope and diversity of this catalyst, several aldehydes containing electron-donating and electron-withdrawing substitutions **7** were reacted with dimedone (5,5-dimethylcyclohexane-1,3-dione, **20**) and 2-naphthol (**23**) to give 12-aryl-8,9,10,12-tetrahydrobenzo[*a*]xanthen-11-one derivatives **27** containing electron-donating and electron-withdrawing substitutions at 100 °C under solvent-free conditions (Scheme 13) [58].

Veisi et al. carried out the synthesis of 2*H*-indazolo[2,1-*b*]phthalazinetrione derivatives **55** and triazolo[1,2-*a*]indazoletrione derivatives **81** using solvent-free reactions between aldehydes **7**, dimedone (**20**), and phthalazine (**54**) or *N*-phenylurazoles (**80**) at 80 °C in the presence of catalytic amount of a mesoporous SBA-15 silica functionalized with sulfonic acid groups (SBA-15-Ph-SO_3_H, **79**). The reactions proceeded well using 5 mol % of the catalyst to form the corresponding products **55** and **81** in good to excellent yields under mild reaction conditions.

**Scheme 14:**
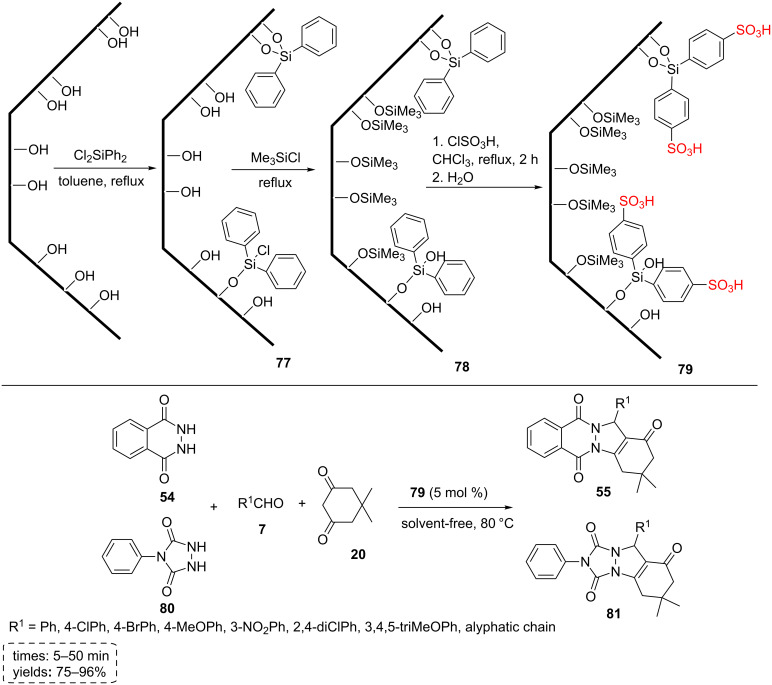
The synthetic route of the SBA-15-Ph-SO_3_H and its catalytic applications for the synthesis of 2*H*-indazolo[2,1-*b*]phthalazinetriones and triazolo[1,2-*a*]indazoletriones.

As a short explanation of the SBA-15-Ph-SO_3_H synthesis, an aqueous solution of Pluronic P123 was added to an aqueous solution of hydrochloric acid. After 2 h, tetraethyl orthosilicate was added and heated to 35 °C for 24 h. The temperature was fixed at 80 °C and the mixture was aged for 24 h without stirring. The resulted material was reacted with dichlorodiphenylsilane in dry toluene under reflux conditions for 12 h to obtain phenyl-modified SBA-15 as a white solid material. In the next step, all -OH groups on the phenyl-modified SBA-15 **77** were protected in dry hexane by the addition of trimethylsilyl chloride. The mixture was refluxed for 8 h to produce trimethylsilylated phenyl-modified SBA-15 **78**. This white solid was reacted with ClSO_3_H to get SBA-15 functionalized with phenyl sulfonic acid groups (SBA-15-Ph-SO_3_H, **79**, Scheme 14). The SBA-15-Ph-SO_3_H catalyst **79** is a hydrophobic nanoreactor solid acid catalyst that presents a series of advantages, such as recyclability, resistant to leaching in organic and aqueous solutions, and stability to water (and also to air and moisture) [59].

In 2015, Rostamnia and co-workers increased the catalytic activity of SBA-15-SO_3_H through hydrophilic/hydrophobic fluoroalkyl-chained alcohols. One of the major drawbacks of the sulfonated mesoporous silica materials is that they are poisoned with water. To increase the hydrophobicity of them, the authors reported some solutions such as confining fluoroalkyl-chain alcohols (R_F_OH) inside them. A range of R_F_OH including trifluoroethanol (TFE), ethanol, hexafluoroisopropanol (HFIP) was explored for tetrasubstituted imidazole synthesis from primary amines, aromatic aldehydes, ammonium acetate, and phenylglyoxal. The TFE-modified SMSM had better behavior than others. To highlight the catalytic activity of the R_F_OH/SBA-15-Pr-SO_3_H, the reaction was also carried out with non-R_F_OH-functionalized catalyst. The SBA-15-SO_3_H containing fluorinated alcohols had more catalytic activity [60].

In an initiative research, Doustkhah and Rostamnia developed a green catalytic system based on SBA-15 mesoporous silica with sulfamic acid content. This heterogeneous Brønsted solid acid was used as an efficient and reusable catalyst for rapid oxidation of a series of aromatic and aliphatic sulﬁdes at room temperature under aqueous medium. The simplicity of the process, chemoselectivity towards sulfoxides, and recyclability at least for eleven runs were the merits of this procedure [61].

**Scheme 15:**
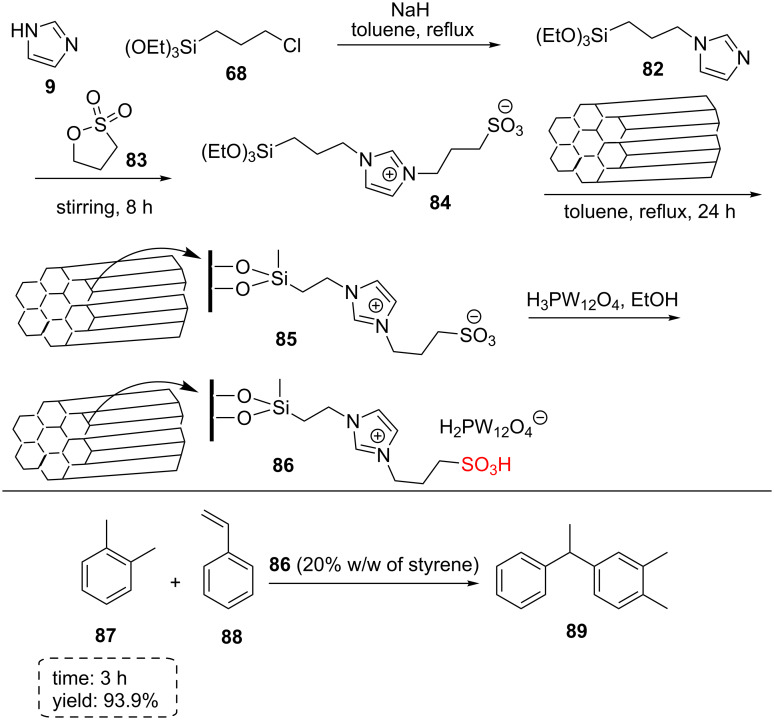
The synthetic route for heteropolyanion-based ionic liquids immobilized on mesoporous silica SBA-15 and its catalytic activity for the alkylation of *o*-xylene with styrene.

Zhou et al. described the immobilizing heteropolyanion-based ionic liquids on mesoporous silica SBA-15. They synthesized intact mesostructures with well-ordered hexagonal arrays of 2D mesoporous channels. *N*-Triethoxysilylpropylimidazole (**82**) was produced by the reaction between imidazole (**9**) and 3-triethoxysilylpropyl chloride (**68**) in the presence of a base (NaH) in toluene as solvent under a nitrogen atmosphere. Then, triethoxysilylpropylimidazole (**82**) and 1,3-propane sultone (**83**) were stirred at 50 °C for 8 h under a nitrogen atmosphere to produce 1-(3-sulfonatopropyl)-3-(3-(triethoxysilyl)propyl)-1*H*-imidazol-3-ium (**84**). A mixture of 1-(3-sulfonatopropyl)-3-(3-(triethoxysilyl)propyl)-1*H*-imidazol-3-ium (**84**) and dry SBA-15 was refluxed in dry toluene for 24 hours under nitrogen atmosphere to produce sulfonated ionic imidazole on SBA-15 **85**. Finally, tungstophosphoric acid (HPW) was added to the dispersed sulfonated ionic imidazole on SBA-15 **85** in deionized water and stirred at 25 °C for 12 h (Scheme 15). The HPW-based ionic liquid immobilized on mesoporous silica SBA-15 **86** displayed excellent utility and reusability for alkylation of *o*-xylene (**87**) with styrene (**88**).

Although the homogeneous HPW displayed very high catalytic activity for the alkylation of *o*-xylene with styrene, it cannot be separated from the reaction mixture. The SBA-15 support itself exhibited no activity for the reaction, but 30% HPW-PMIMPS-SBA-15 material produced the highest yield and showed good selectivity. Decreasing and increasing in the amount of HPW on silica decreased the yield of reaction [62].

**Scheme 16:**
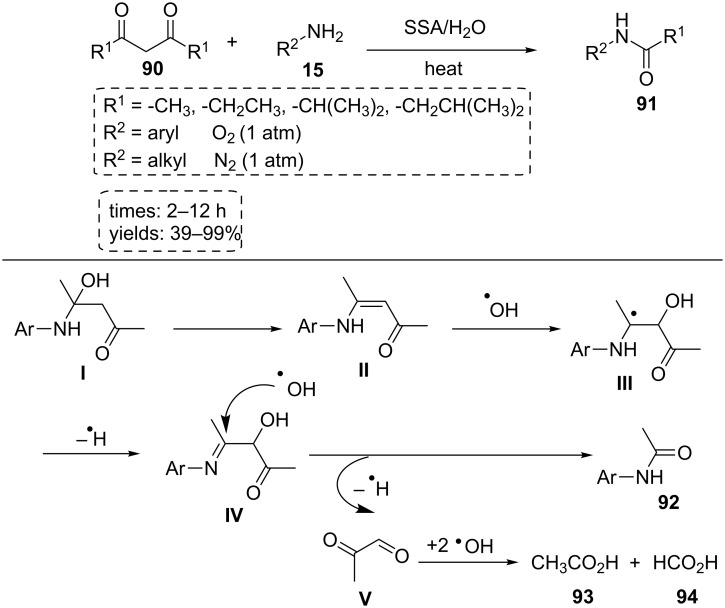
Some mechanism aspects of SSA catalyst for the protection of amine derivatives.

The silica sulfuric acid (SSA) catalyst was synthesized by the treatment of silica gel with sulfuryl chloride under room temperature stirring. The catalyst was used in the acylation of amines with 1,3-diketones via C–C bond cleavage. Various protected aniline derivatives were obtained by the solvent-free reaction of anilines with 1,3-diketones at 120 °C or 140 °C in the presence of 2 equiv of water under 1 atm O_2_ atmosphere (Scheme 16). The probable reaction pathway for this reaction is shown in Scheme 16. Arylamine **15** attacks the activated carbonyl of acetylacetone **90** to form enamine intermediate **II**. In the next step, hydroxyl radicals are produced through activation of molecular oxygen in the presence of SSA. Intermediate **III** is created via the addition of a hydroxyl radical to enamine **II**. This intermediate loses hydrogen radical to form intermediate **IV**. Finally, this intermediate is split by a nucleophilic attack of hydroxyl radicals to afford byproducts (including acetic acid, acetanilide, and formic acid) and desired product. The proposed mechanism was confirmed by EPR spectrum. The SSA catalyst is an inexpensive and reusable solid acid catalyst [63].

### 4. Synthetic strategies of carbon-based materials and functionalized carbon-based materials containing sulfonic acid groups as a diverse class of catalysts and their uses in organic reactions

Carbon-based materials containing sulfonic acid groups [33] have been used as novel, efficient, and reusable catalysts, due to their ability to catalyze different chemical processes in industry and laboratories. There are many reports on the carbon-based materials containing the sulfonic acid group, but the future advances of this field will depend on the better understanding of all aspects of their synthetic routes and catalytic applications [64]. The various properties including tunable porosity, stability, and surface chemistry of the carbon-based materials make the carbon-based materials appropriate for use in many catalytic transformations.

In this regard, different functionalized carbon-based materials containing sulfonic acid groups as novel acid catalysts were employed in different industrial and synthetic reactions which some of them were mentioned below.

**Scheme 17:**
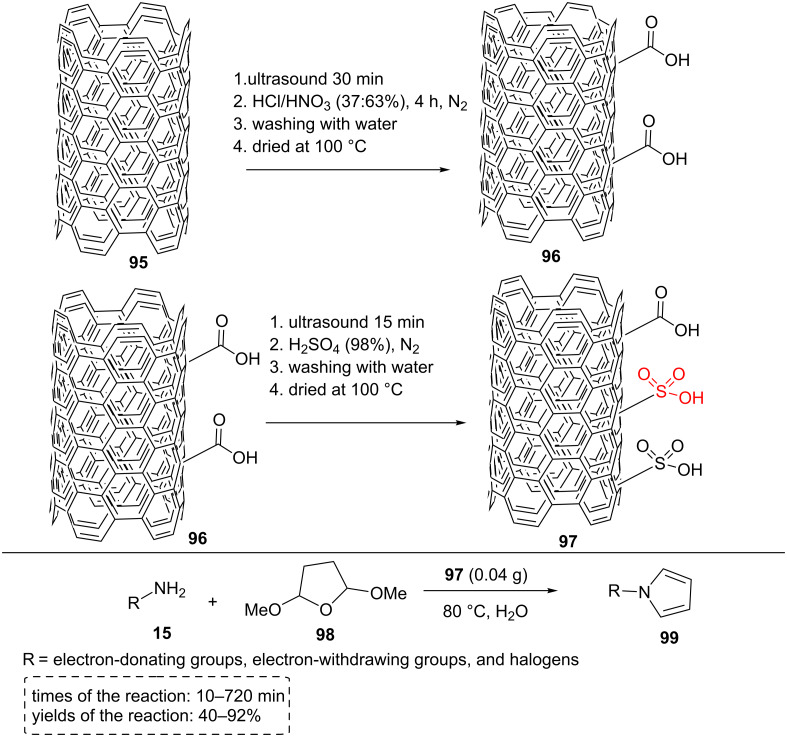
The synthetic route for MWCNT-SO_3_H and its catalytic application for the synthesis of N-substituted pyrrole derivatives.

Sulfonated multi-walled carbon nanotubes (MWCNT-SO_3_H) can be created following the strategy illustrated in Scheme 17. Supported sulfonic acid is generally prepared in several steps: (a) sonication of MWCNTs for 30 min, (b) addition of sonicated MWCNTs to another flask containing HNO_3_ and HCl with stirring at 80 °C for 4 h to form MWCNTs-COOH material **96**, (c) sonication of MWCNTs-COOH for 15 min, (d) addition of H_2_SO_4_ to a set-up at 250–270 °C for 20 h, (e) after filtration, washing, and drying, MWCNTs-SO_3_H composite **97** was achieved (Scheme 17).

N-Substituted pyrroles **99** were obtained in good to excellent yields (40–92%) via a simple and green reaction between 2,5-dimethoxytetrahydrofuran (**98**) and primary amines **15** in water media at 80 °C using MWCNTs-SO_3_H composite **97** as the efficient and heterogeneous catalyst. The reaction was also performed under the same conditions using different catalysts including Fe_3_O_4_, CuFe_2_O_4_, ZnS nanoparticles, TiO_2_, MWCNTs, MWCNTs/H_2_SO_4,_ MWCNTs–COOH, and Ph–SO_3_H, but the products were not achieved in appropriate yields and times.

The easy preparation of the catalyst, short reaction times, easy handling, low-cost procedure, good to excellent reaction yields, and eco-friendly are some of the advantages of this study. The reusability of the catalyst is a very significant feature. In this case, the catalyst was filtered, washed with chloroform and ethanol, and finally dried at 100 °C for 24 h. The catalyst was reused for four runs with good results (the yields of products ranged from 40 to 92%) [65].

**Scheme 18:**
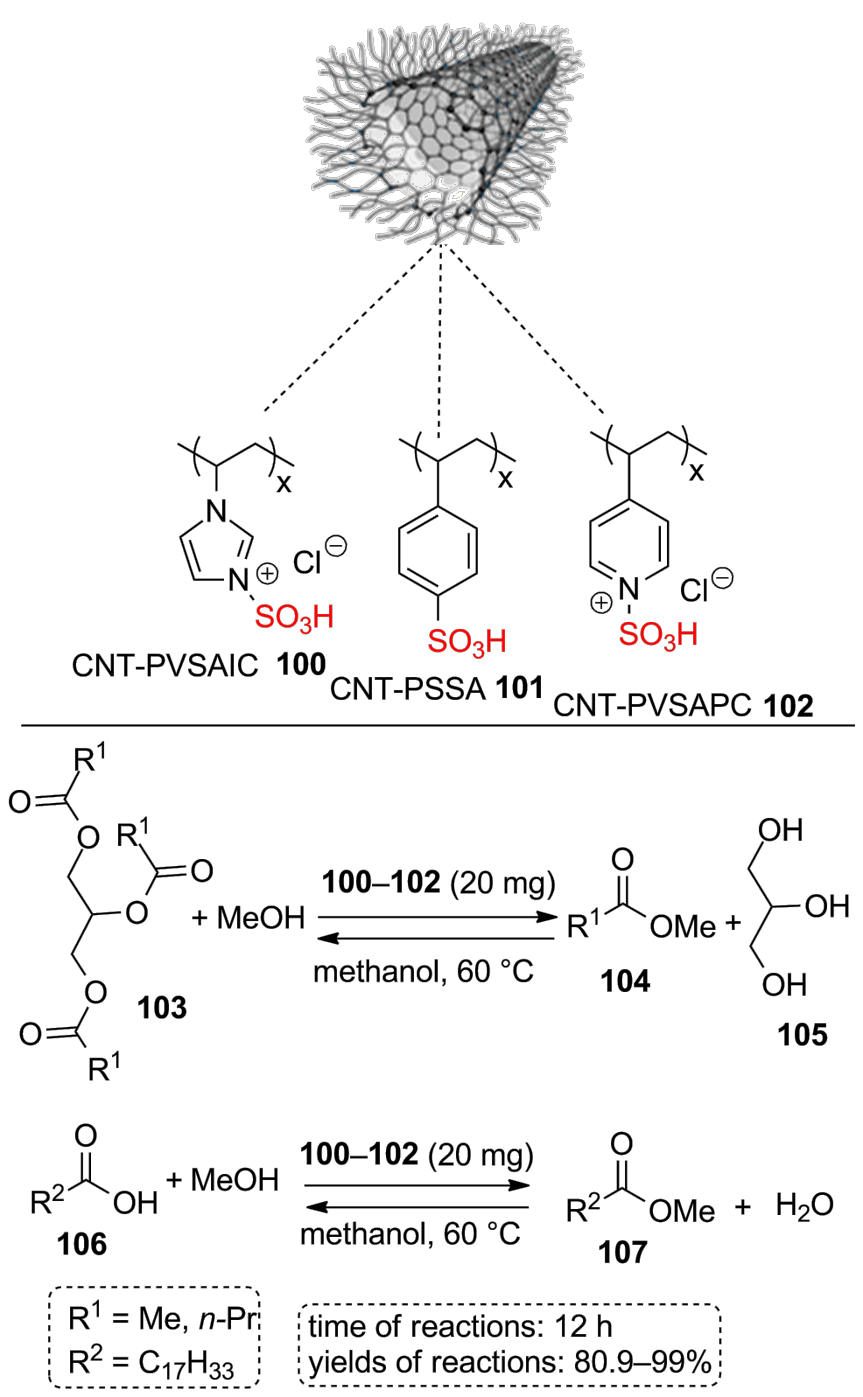
The sulfonic acid-functionalized polymers (P-SO_3_H) covalently grafted on multi-walled carbon nanotubes and their catalytic applications for transesterification of triglycerides with methanol and the esterification of oleic acid with methanol.

A series of sulfonated polymers covalently grafted on multiwall carbon nanotubes (MWCNTs) composite materials such as poly(1-vinyl-3-sulfo-1*H*-imidazolium chloride) grafted on MWCNT **100**, poly(4-styrenesulfonic acid) grafted on MWCNT **101**, and poly(4-vinyl-1-sulfo-pyridinium chloride) grafted on MWCNT **102** (CNT-PVSAIC, CNT-PSSA, and CNT-PVSAPC, respectively) was prepared (Scheme 18). Obtained sulfonated polymer-carbon nanotubes composites (CNT-P-SO_3_H) **100**–**102** were described as outstanding catalysts for liquid phase transesterification of triglycerides **103** with methanol. The catalysts were also used for the esterification of oleic acid (**106**) with methanol. The important feature of this study is that these reactions are considered as typical model reactions in biodiesel production. To highlight the effect of acid groups, authors investigated the imidazolyl and pyridinyl polymers grafted on MWCTs for the transesterification of triglyceride **103** and low desired product (ranging from 12.3 to 15.1%) was obtained under same conditions. This may be related to Brønsted basicity of imidazolyl and pyridinyl groups. In addition to the catalytic role of well-extended P-SO_3_H coating over the external surface of the CNT, even the mesoporous structure of the support may play a role in catalysis [66].

**Scheme 19:**
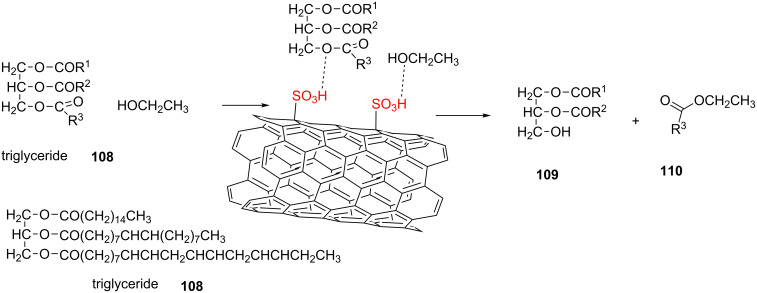
The transesterification reaction in the presence of S-MWCNTs.

In the next example, sulfonated multi-walled carbon nanotubes were reported as a catalyst to produce fatty acid ethyl ester (biodiesel production). In this case, triglycerides **108** and ethanol were absorbed through the interaction between the acid sites on MWCNTs and the oxygen atom of substrates. The oxygen of ethanol likely attacks the carbon of the carbonyl group to produce the final product (Scheme 19). It should be noted that the reaction proceeded well in the presence of 3.7 wt % of the catalyst to produce a high yield of the desired product (overall conversion of 97.8%) in ethanol at 150 °C for 1 h [67].

The hypercrosslinked supermicroporous polymer (HMP-1, **113**) was also designed and prepared by iron(III) chloride catalyzed Friedel–Crafts alkylation of carbazole (**111**) with α,α′-dibromo-*p*-xylene (**112**). In the next step, HMP-1 (**113**) was sulfonated by Cl-SO_3_H to form HMP-1-SO_3_H material **114**. The α,α′-dibromo-*p*-xylene (**112**) was chosen as a linker because of containing the benzene rings for post-synthetic functionalization. HMP-1 (**113**) and HMP-1-SO_3_H (**114**) were confirmed by IR spectroscopy. The peak corresponding to the C–Br bond was not detected in the FTIR spectrum of HMP-1. The peaks at 2900 cm^−1^ and 3400 cm^−1^ were the evidence of phenylic C–H bond and N–H stretching of carbazole, respectively. Peaks at 1022 cm^−1^ and 1039 cm^−1^ corresponded to additional crosslinking during the sulfonation process. The catalyst was investigated by BET, SEM, TEM, and TGA-DTA, as well. The BET surface area decreased from 913 m^2^ g^−1^ to 346 m^2^g^−1^ during the sulfonation process. The SEM images of HMP-1 (**113**) and HMP-1-SO_3_H (**114**) showed spherical particles. According to the TGA-DTA plot, HMP-1-SO_3_H (**114**) is less stable than HMP-1 (**113**).

**Scheme 20:**
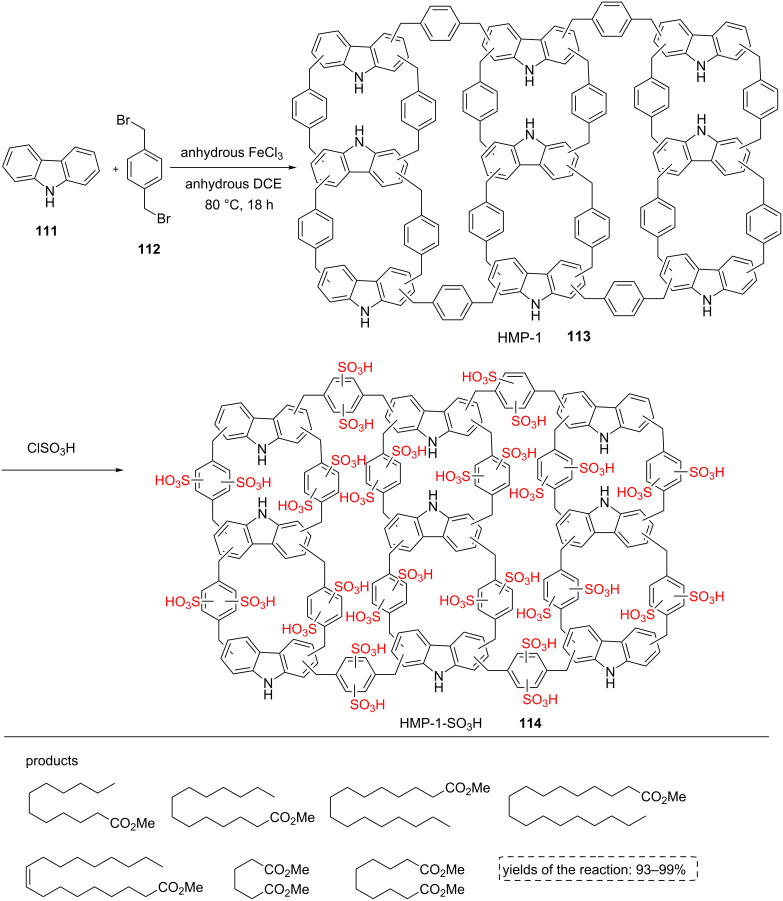
The synthetic route for the new hypercrosslinked supermicroporous polymer via the Friedel–Crafts alkylation reaction of carbazole with α,α-dibromo-*p*-xylene and its catalytic application in biodiesel synthesis.

The resulted catalyst was tested in biodiesel synthesis at room temperature. The esterification of long chain-free fatty acids with methanol was performed well for 10–12 h (Scheme 20). To check the effect of acid groups, HMP-1 (**113**) was also used as a catalyst for the reaction and low yields of corresponding products were obtained [68].

**Scheme 21:**
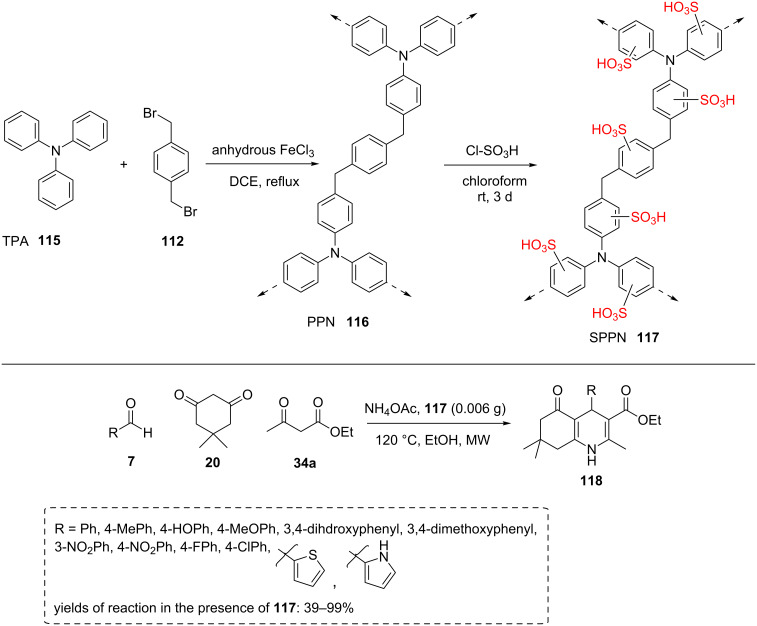
The synthetic route for a new microporous copolymer via the Friedel–Crafts alkylation reaction of triphenylamine with α,α-dibromo-*p*-xylene and its catalytic application in multicomponent synthesis.

In another study, a new microporous copolymer synthesized by Friedel–Crafts alkylation of triphenylamine (**115**) with dibromo-*p*-xylene **112** was prepared (Scheme 21). After the sulfonation process, the resulted material has been employed as a heterogeneous, reusable, and environmentally benign catalyst in the multicomponent synthesis of polyhydroquinoline derivatives **118** under microwave irradiation. Several substituted aldehydes **7** with dimedone (**20**), acetoacetate ester **34a**, and ammonium acetate in ethanol under microwave irradiation were reacted to produce corresponding products in high yields. In addition to the catalytic role of strong acid strength, the high surface area may play a role in catalysis. The catalyst was reused up to five cycles [69].

**Scheme 22:**
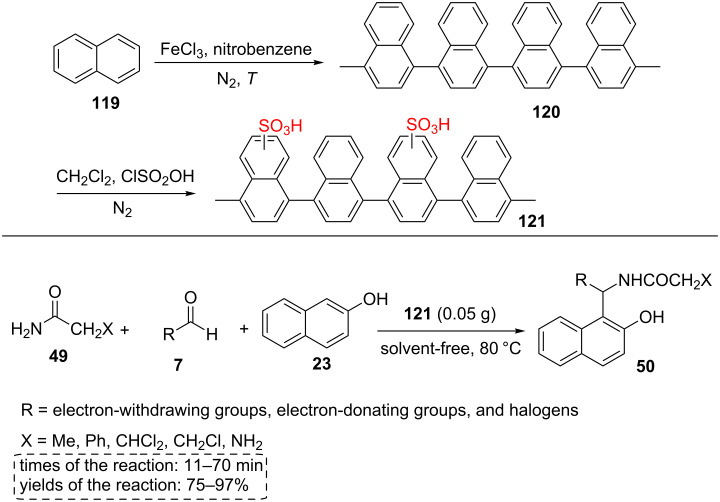
The synthetic route for sulfonated polynaphthalene and its catalytic application for the amidoalkyl naphthol synthesis.

Pourmousavi et al. [70] found that sulfonated polynaphthalene **121** synthesized from naphthalene (**119**) shows high catalytic activities in the one-pot preparation of amidoalkyl naphthols **50**. The sulfonated polynaphthalene can be achieved in two steps: (a) polymerization of naphthalene (**119**) in nitrobenzene using FeCl_3_ as reagent (b) sulfonation of polynaphthalene (**120**) with chlorosulfonic acid in dichloromethane (Scheme 22).

Amidoalkylnaphthol derivatives **50** were synthesized using catalyst **121** under thermal solvent-free conditions with good to excellent yields (75–97%) for short reaction times (11–70 min, Scheme 22). The three-component reaction tolerated one-substituted arylaldehydes including *p*-Cl, *o*-Cl, *o*-NO_2_, *m*-NO_2_, *p*-NO_2_, *o*-OMe, *p*-OMe, *p*-OH, and *p*-F as well as two- or three-substituted arylaldehydes including 2,4-dichloro, 2,4-dimethoxy, 3,4-dimethoxy, and 3,4,5-trimethoxy. The catalyst was used up to four cycles under the optimized conditions [70].

**Scheme 23:**
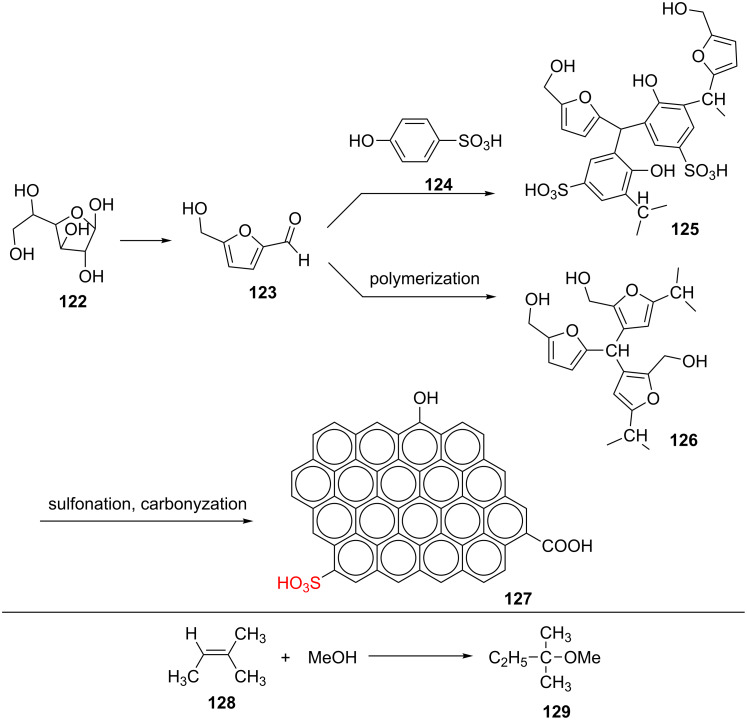
The synthetic route of the acidic carbon material and its catalytic application in the etheriﬁcation of isopentene with methanol.

A new strategy was proposed for the synthesis of a novel sulfonated carbon catalyst **127** using the reaction of 5-(hydroxymethyl)furfural (**123**) with 4-hydroxybenzenesulfonic acid (*p*-HBSA, **124**). As a simple and brief explanation of the sulfonated carbon synthesis, 5-(hydroxymethyl)furfural (**123**) and *p*-HBSA (**124**) were dissolved in deionized water to produce a clear brownish red solution. In continuation, the solution was heated at 358 K and stirred for 2 h. After evaporation of water, a black viscous paste was created. The paste was heated at 303 K for 1 h to produce a black solid. The solid was then washed, filtered, and dried at 353 K. To carbonize and sulfonize the solid, it was heated in concentrated sulfuric acid at 443 K for 12 h. Finally, the carbonized sample was washed and dried at 353 K overnight. The final material was evaluated as a recoverable catalyst with strong surface acid sites for the etheriﬁcation of isopentene (**128**) with methanol (Scheme 23). In this regard, a mixture of isopentene (10 g), methanol (4.57 g), toluene as solvent (35.43 g), and catalyst (0.5 g) was placed in an autoclave equipped with a magnetic stirrer. After sealing and purging with N_2_, it was heated to 353 K for 20 h under stirring. The catalytic activity of the catalyst was studied for three cycles of the reaction and isopentene successively converts to the product in the same yields (55.2, 55.9 and 54.3%, respectively) [71].

Another research reported for sulfonated carbon material is using low-cost resorcinol (**130**) and formaldehyde (**131**) solution. In this case, resorcinol (**130**) was added to a stirring solution of aqueous ammonia solution, absolute ethanol, and deionized water. In the next step, formaldehyde (**131**) solution was added and stirred for 24 h at 30 °C. The resulted solution was placed in a Teﬂon-sealed autoclave and heated at 100 °C for 24 h. Subsequently, the product was centrifuged, washed, and dried. The carbon nanospheres material **132** was obtained by a carbonization process at 400 °C for 2 h in an N_2_ atmosphere [72–73].

**Scheme 24:**
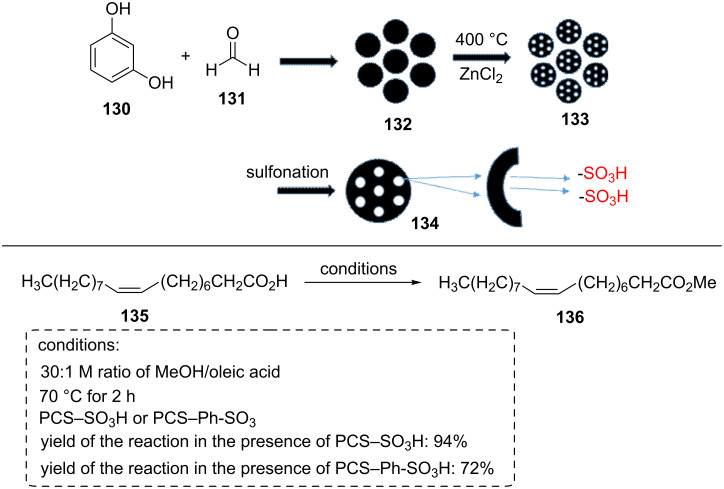
The synthetic route of the acidic carbon materials and their catalytic applications for the esterification of oleic acid with methanol.

This material was dispersed into an aqueous solution containing zinc chloride and stirred for 3 h. After completely evaporation of the aqueous solution, ZnCl_2_-impregnated RF resin spheres were obtained. Next, the ZnCl_2_-impregnated RF resin spheres were activated at 400 °C for 2 h in an N_2_ atmosphere. Subsequently, the resulted material was washed with HCl solution and distilled water and then dried under vacuum at 80 °C for 10 h to form porous carbon nanospheres material **133**. Finally, the material was sulfonated by concentrated sulfuric acid or *p*-toluenesulfonic acid (Scheme 24) [73].

The catalytic applications of these sulfonated carbon catalysts were investigated in the esterification of oleic acid with methanol (with different ratios of MeOH/oleic acid) at 70 °C for 2 h. Theoretically, the esterification process requires one equivalent of oleic acid and one equivalent of methanol to achieve one equivalent of the desired product. But, the esterification is a reversible process, and thus needs the excessive amount of methanol to increase the conversion of reactants into products. So high yields were obtained by the 30:1 molar ratio of MeOH/oleic acid.

**Scheme 25:**
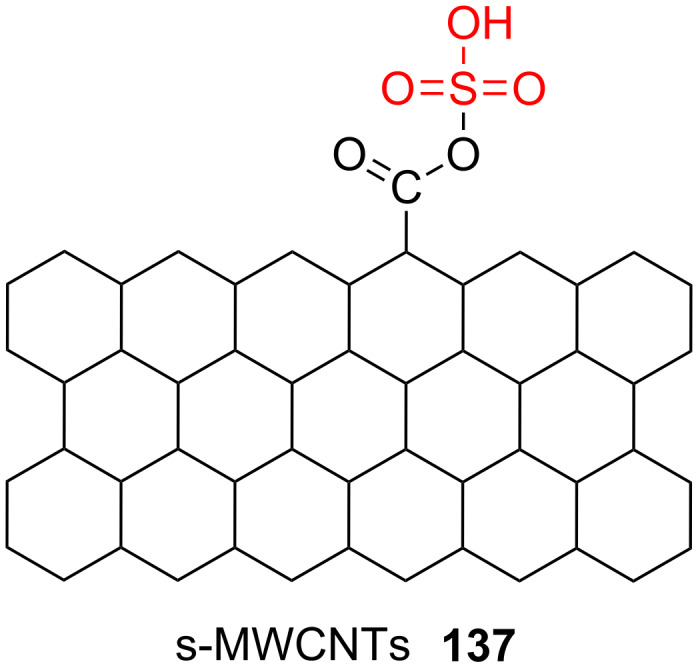
The sulfonated MWCNTs.

It is well known that grafting MWCNTs with -SO_3_H functions is very useful for activation of catalysts. On the other hand, the most common technique to sulphonate these materials is through thermal treatment by concentrated sulfuric acid. These procedures are time-consuming and energy intensive, as well. Zou et al. reported an effective strategy for acid-free sulfonation of MWCNTs using the combination of ultrasonication and heating of the mixture of MWCNTs-COOH and (NH_4_)_2_SO_4_ solution. After washing the mixture with distilled water, the final product was produced and defined as s-MWCNTs **137** (Scheme 25). In the next step, the type of acid sites on this solid product was identified via pyridine-FTIR spectroscopy. The FTIR spectrum of s-MWCNTs **137** before pyridine adsorption showed no sharp signal, but the FTIR spectrum of s-MWCNTs after pyridine adsorption showed some peaks at 1646, 1626, 1549 and 1476 cm^−1^. The peaks at 1646, 1626, and 1549 cm^−1^ could be due to the vibration of pyridinium (PyH^+^) species, corresponding to the existence of Brønsted acid sites on the s-MWCNTs **137**. The peak at 1476 cm^−1^ was also labeled to the coordination of electron pair in the nitrogen orbital of pyridine to Brønsted acid sites. No IR signal relating to the Lewis acid sites was detected at 1455 cm^−1^, as well. Finally, the s-MWCNTs **137** were used in the esteriﬁcation of palm fatty acid distillate (PFAD) with methanol. The esteriﬁcation of palm fatty acid with methanol was performed under following conditions: a pressure of 10 bar, a reaction temperature of 170 °C, a reaction time of 3 h, 20:1 molar ratio of MeOH/palm fatty acid distillate, and 2 wt % of catalyst [74]. An important thing to note is that the s-MWCNTs should be stirred in methanol for 10 min before use in the reaction. This causes that the tendency of adsorption of PFAD on active sites was decreased and consequently the catalyst will not be deactivated.

**Scheme 26:**
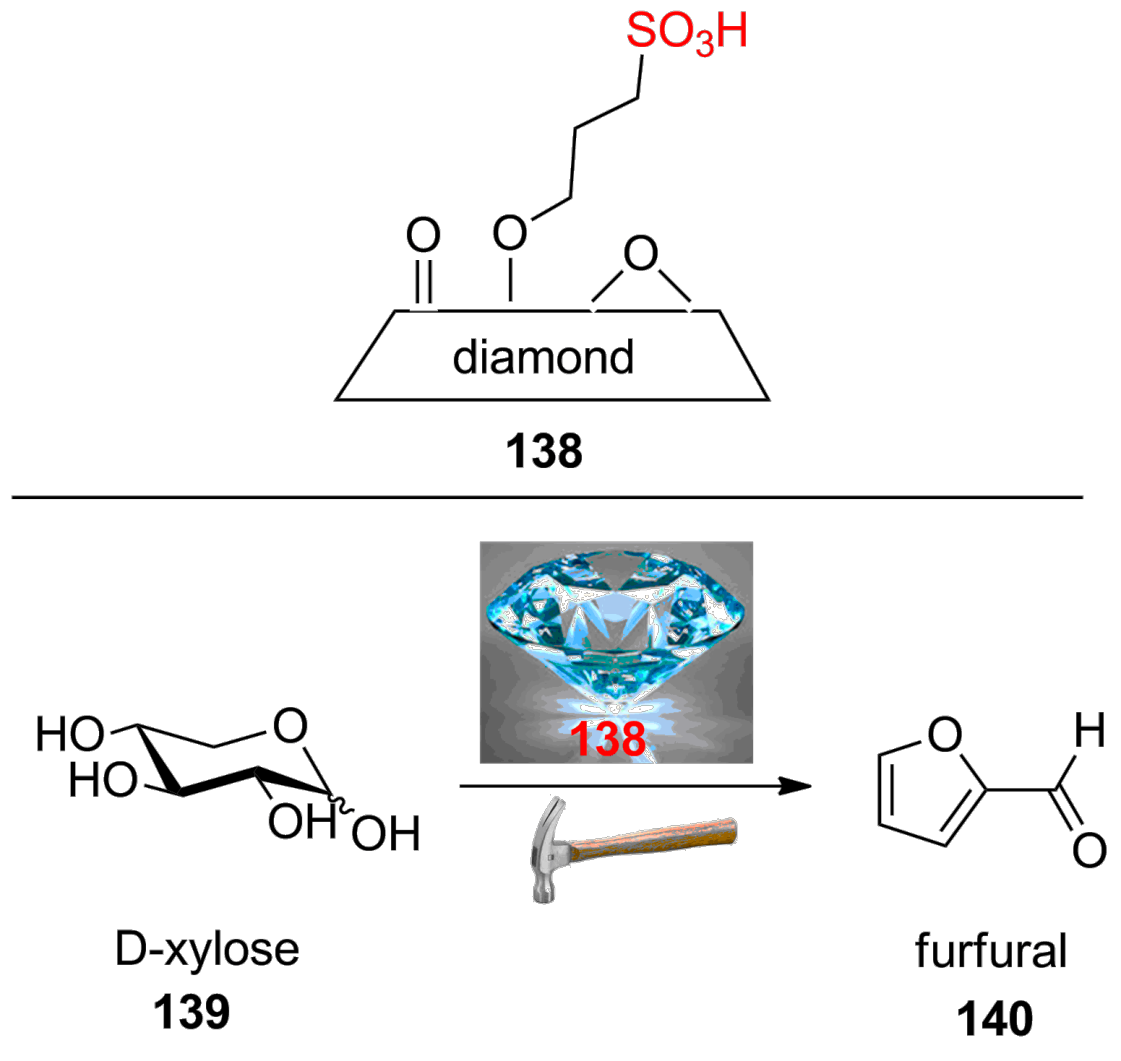
The sulfonated nanoscaled diamond powder for the dehydration of D-xylose into furfural.

A covalently grafting modiﬁed nanoscaled diamond powder with 1,3-propanesultone (Scheme 26) **138** exhibited excellent catalytic activity for esteriﬁcation under atmospheric conditions and conventional heating [75]. The catalyst showed excellent catalytic activity in the dehydration of D-xylose (**139**) into furfural (**140**) as an industrial platform molecule as well as the production of ethylacetate from ethanol and acetic acid [76]. The dehydration of D-xylose (**139**) into furfural (**140**) was performed in water-CPME (1:3, v/v) and heated in a commercial monowave microwave oven in the presence of 10 wt % of **138** for 50 min. A maximum furfural yield of 76% was obtained.

**Scheme 27:**
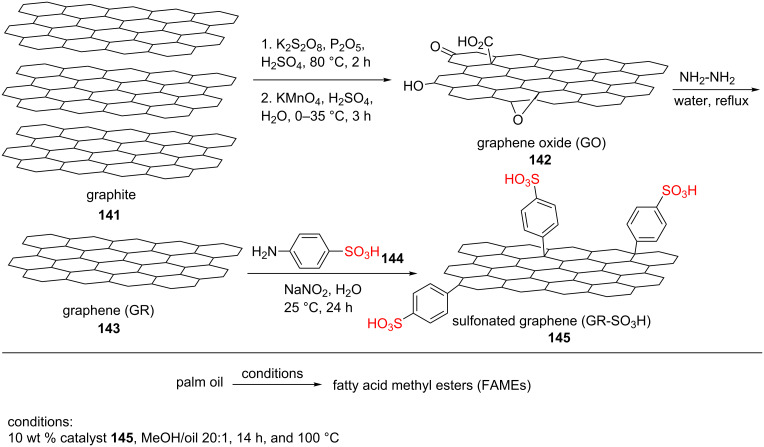
The synthetic route and catalytic application of the GR-SO_3_H.

A new sulfonated graphene catalyst GR-SO_3_H (**145**) was prepared in some steps. A mixture of graphite powder (**141**), potassium persulfate, phosphorus pentoxide, and sulfuric acid was heated at 80 °C for 2 h. The resulted solid was ﬁltered and washed with water, methanol, and ether. Then, the obtained black paste was dried. After mixing the resulting material with sulfuric acid at 0 °C, potassium permanganate was added and stirred at 35 °C for 2 h. In the next step, the reaction mixture was cooled to 0 °C. Hydrogen peroxide (30%) in deionized water was added to the reaction mixture. The solid was gathered by centrifugation, washed with deionized water, methanol, and ether, and then dried at 40 °C under vacuum. The resulting brown solid material was named as graphene oxide (GO, **142**). In continuation, a solution of 5% sodium carbonate was added to the sonicated GO (**142**) in deionized water so that the pH was increased up to 9–10. 64% hydrazine hydrate was added as well. Then, the reaction mixture was heated to reﬂux for 24 h. After cooling down to room temperature, the solution was ﬁltered, washed with 1 N HCl and acetone, and then dried. The resulted material was named as graphene (GR, **143**). In order to sulfonate the GR (**143**), sodium nitrite and sulfanilic acid (**144**) were added to a sonicated solution of GR (**143**). Finally, the solution was ﬁltered, washed with 1 N HCl and acetone, and dried to produce GR-SO_3_H (**145**, Scheme 27). The effect of different parameters including reaction temperature, catalyst loading, reaction time, and methanol-to-oil molar ratio was investigated for the transesteriﬁcation of palm oil with methanol into biodiesel. The results showed that the reaction proceeded well by the 20:1 molar ratio of MeOH/oil in the presence of 10 wt % catalyst at 100 °C for 14 h.

The progression of this reaction was studied by ^1^H NMR in CDCl_3_. The ^1^H NMR spectrum of palm oil exhibited some peaks at 4.10–4.32 and 5.31–5.35 ppm for the glycerol scaffold and olefinic protons, respectively. Biodiesel formation was approved through the invisibility of the protons of the glycerol scaffold and appearance of a single peak at 3.63 ppm related to the methyl esters of fatty acids. The catalyst exhibited excellent catalytic activity and reusability for the reaction. The heterogeneous GR-SO_3_H (**145**) displayed a high thermal robustness, as well [77].

## Conclusion

A comprehensive and systematic overview was presented of recent researches that focused on the design, synthesis, and catalytic applications of sulfonated organic materials, sulfonated silica materials, and sulfonated carbon materials as novel catalysts with several features. The efforts to design and preparation of different type of sulfonated catalysts not only focus on the laboratory scale but also on an industrial scale. Researchers are trying to reduce costs of catalyst preparation and regeneration. We believe this review article is valuable for the future design of highly active acidic catalysts.
